# The arms race in bacteria-phage interaction: deciphering bacteria defense and phage anti-defense mechanisms through metagenomics

**DOI:** 10.3389/fmicb.2025.1687307

**Published:** 2025-10-15

**Authors:** Siyuan Zhang, Mengqi Chu, Xumei Sun

**Affiliations:** ^1^School of Marine Sciences, Ningbo University, Ningbo, China; ^2^Key Lab of Artificial Organs and Computational Medicine, Institute of Translational Medicine, Zhejiang Shuren University, Hangzhou, Zhejiang, China

**Keywords:** bacteriophages, interactions, co-evolution, immune defense, metagenomics

## Abstract

Bacteriophages are viruses that specifically infect bacteria and co-evolve with their hosts through mutual interactions. They represent one of the most significant drivers of microbial diversity, influencing its evolution, generation, and maintenance. To counter bacteriophage infection, bacteria have developed sophisticated immune systems, including both passive adaptations, such as inhibiting phage adsorption and preventing DNA entry, and active defense systems such as restriction-modification systems and CRISPR-Cas systems. The ongoing arms race between bacteriophages and bacteria has left distinct evolutionary signatures in their genomic sequences. Advances in large-scale genomic and metagenomic sequencing technologies, coupled with bioinformatics approaches, have greatly enhanced our understanding of bacteria-phage interaction mechanisms, driving progress in bacteriophage biology. This review systematically analyses the diverse immune strategies bacteria employ against phage infection, elucidates the coordination and interrelationships among different anti-phage mechanisms, and highlights potential directions for future research.

## 1 Introduction

As viruses that specifically infect and lyse pathogenic bacteria, bacteriophages exhibit high specificity and offer a promising alternative to combat antibiotic-resistant bacterial infections (Benler et al., 2018; Cano et al., 2021). However, due to the co-evolutionary arms race between bacteriophages and their bacterial hosts, phage therapy resistance frequently emerges in pathogenic bacteria (Bernheim and Sorek, 2020; Diercks et al., 2025; Roch et al., 2025). Although clinical applications of phage therapy have incorporated strategies such as multi-phage cocktails to mitigate resistance (Weiner et al., 2025), these approaches remain insufficient. Therefore, a deeper understanding of the underlying interaction mechanisms between bacteriophages and their host bacteria is essential. Over long-term co-evolution, bacteriophages impose strong selective pressures on bacterial populations, driving the development of complex immune systems that enable bacteria to resist or evade phage infection (Stern and Sorek, 2011; Arnold et al., 2022). These systems collectively define bacterial immunity—the ability of bacteria to maintain cellular integrity and ensure survival under environmental stress through precisely regulated mechanisms (Dy et al., 2014). Upon infection, bacteriophages progress through their replication cycle through sequential stages including adsorption, invasion, uncoating, biosynthesis, lysis, and release (Olszak et al., 2017; Vendrell-Fernández et al., 2025). Throughout evolutionary history, bacteria have developed distinct immune strategies targeting each stage of this cycle (Labrie et al., 2010). Traditionally, the analysis of bacteria-phage interactions has relied on virus-host culture systems, which are considered the gold standard for detecting the presence of such interactions (Quince et al., 2017). However, this method is labor-intensive, low-throughput, and often limited by the inability to culture many phages and bacterial strains *in vitro*. Consequently, a comprehensive understanding of phage-bacteria interactions using solely experimental approaches remains challenging. With the rapid development of molecular technologies, high-throughput metagenomic sequencing has emerged as a powerful tool (Taş et al., 2021). This technique enables direct sequencing and analysis of all microorganisms and their genomes within environmental samples without the need for purification, isolation, or cultivation (Carter et al., 2023). Compared to traditional culture-based methods, it offers higher sensitivity and accuracy, facilitating the rapid identification of novel microbial species and revealing an unprecedented view of microbial ecosystems (Johansen et al., 2022; Sun et al., 2023). Therefore, metagenomic sequencing provides a novel and effective approach for investigating phage-bacteria interactions.

The rise of metagenomic technologies has significantly enhanced our understanding of microbial diversity and its spatiotemporal dynamics across various ecosystems—from the deep sea to soil and even the gastrointestinal tracts of mammals (Shkoporov et al., 2019; Zheng et al., 2021; Muscatt et al., 2023; He et al., 2024). Within these ecosystems, bacteriophages and bacteria represent the most abundant and diverse biological entities. As bacteria-specific viruses, bacteriophages coexist with bacteria through dynamic interactions, serving as a key evolutionary force that shapes microbial communities and plays a central role in generating and maintaining of microbial biodiversity (Koskella and Brockhurst, 2014; Laanto et al., 2017). Beyond advancing our understanding of microbial ecology and evolution, studying phage-bacteria interactions also provides insights into the role of bacteriophages in bacterial virulence evolution and their potential clinical applications (Levin and Bull, 2004; Scanlan and Buckling, 2012). During their coevolution, bacterial traits such as growth, metabolic activity, pathogenicity, antibiotic resistance, and interspecies competition may all be influenced by bacteriophage infection (Wein and Sorek, 2022). Conversely, bacteriophages rely on bacterial hosts for reproduction and replication, continuously undergoing mutation and recombination to adapt to diverse host environments (Miura and Tomizawa, 1970; Rohwer, 2003). As a result, the characteristics of their interactions are preserved within the genomes of both bacteriophages and bacteria (Amitai and Sorek, 2016). Currently, researchers have developed numerous accurate, robust, and scalable algorithms aimed at predicting bacteriophage-bacteria interactions (Hannigan et al., 2018). These algorithms enable the systematic identification of novel and efficient bacterial immune systems from large-scale sequencing data by leveraging coevolutionary features between bacteriophages and bacteria. This approach enhances our understanding of bacterial immune defense mechanisms against bacteriophages, as well as the counter-mechanisms employed by bacteriophages to evade bacterial immune defenses, thereby facilitating comprehensive and systematic studies of their interactions.

## 2 Bacteriophages specifically regulate bacterial hosts

Bacteriophages are ubiquitous in natural environments and can be detected wherever bacterial hosts are present, making them a major force in shaping microbial community composition (Naureen et al., 2020). They exhibit remarkable diversity and can be classified based on various characteristics. Their genetic material may consist of RNA or DNA, which can be either double-stranded or single-stranded (Khan Mirzaei et al., 2021). Morphologically, their tails can be long and contractile or short (Dion et al., 2020). To date, double-stranded DNA bacteriophages have been the most extensively studied, with tailed bacteriophages accounting for over 90% of all described phages (Earnshaw and Casjens, 1980). This bias may stem from the historical reliance on isolation and culture techniques for phage identification. Recently developed metagenomic sequencing methods that do not require isolation or culture have uncovered numerous lineages of non-tailed double-stranded DNA bacteriophages and distinct subfamilies of single-stranded DNA bacteriophages (Waller et al., 2014). These discoveries have significantly expanded our understanding of phage diversity, thereby enhancing our comprehension of the interaction dynamics between bacteriophages and bacteria.

**Figure 1:**
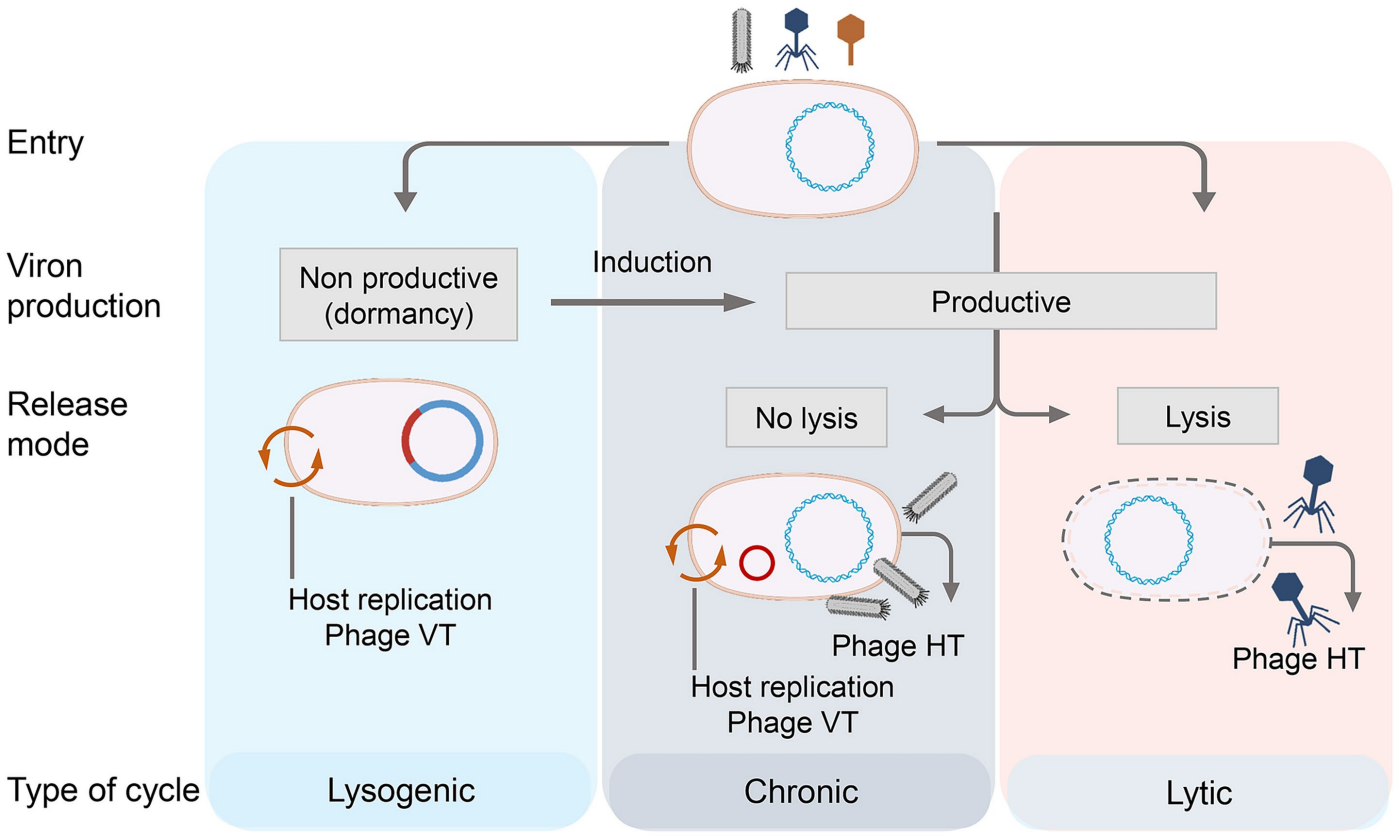
Life history of phage and the way that the phage acts on the host bacteria. Bacteriophage life cycles: lysogenic, chronic, and lytic. The lysogenic cycle is characterized by viral genome integration and dormancy, with no virion production. The chronic cycle enables continuous release of new phages without immediate host cell lysis. The lytic cycle results in host cell lysis and the release of progeny phages. Each cycle includes key stages: viral entry, replication and virion assembly, and release mechanism.

Bacteriophages can also be categorized according to their life cycles, which include chronic, lytic, and lysogenic types (Erez et al., 2017) (Figure 1). Bacteriophages specifically attach to receptors on the bacterial surface and inject their genetic material into the host cell (Sanchez-Torres et al., 2024). They then utilize host-derived enzymes to replicate their genetic material and produce progeny phages. In the chronic life cycle, progeny phages continuously assemble and are released without causing host cell lysis. In the lytic cycle, after injecting nucleic acids into the host cell, the bacteriophage rapidly synthesizes early proteins that degrade the host’s genetic material and hijack cellular processes. It then uses the host’s cellular machinery to synthesize the remaining structural proteins required for assembling new phage particles, and the newly replicated genetic material is packaged into the virion (Wang et al., 2024). Throughout the lytic process, bacteriophage-encoded enzymes progressively degrade the host cell, ultimately leading to its lysis and the release of progeny phages into the environment. In the lysogenic cycle, the bacteriophage integrates its genetic material into the host genome via a phage-encoded integrase, rather than killing the host. The integrated phage genome is then passively replicated along with the host genome (Howard-Varona et al., 2017).

Although well-established laboratory models accurately describe phage replication in these three infection cycles, increasing evidence suggests that these models may not fully capture the complexity of bacteria-phage interactions in natural settings (Álvarez-Espejo et al., 2024). Some studies propose that phage infection strategies may be environmentally responsive rather than fixed, transitioning from productive infections that generate new phage particles to persistent, non-productive infections that do not produce new phages but still propagate the phage genome within the bacterial population (Gaborieau et al., 2024). Lysogenic and lytic phages are not uniformly distributed across ecosystems, and this distribution may be influenced by host density. Under conditions where hosts can proliferate and reach high densities, lytic replication is typically favored. In contrast, when host abundance is low, lysogenic replication tends to dominate (Hobbs and Abedon, 2016).

Phages interact with bacterial hosts through multiple mechanisms and exert significant influences on them. The most prevalent mechanism involves regulating the composition and abundance of host bacteria, thereby affecting the diversity of bacterial communities (Federici et al., 2021; Sun et al., 2024a). The impact of lytic phages on bacterial cell density and community diversity may be partially attributed to cell lysis, which exerts both direct effects on bacterial populations and indirect effects on competition among bacterial strains and species (Morella et al., 2018). Phages not only alter the composition of bacterial populations but also serve as a crucial driving force for bacterial evolution. This evolutionary pressure arises from the intense selective forces exerted by phages through their predatory behavior and their ability to integrate into the bacterial genome as prophages via mechanisms such as lysogeny, transduction, and host gene disruption (Chee et al., 2023). The regulatory role of phage communities on their bacterial hosts can occur through multiple pathways, including distinct replication cycles (lysogenic or lytic), the carriage of unique genetic elements that enhance host adaptability (e.g., virulence factors or antibiotic resistance genes), and the modulation of host mutation rates (Taylor et al., 2019; Pfeifer et al., 2022). Collectively, these mechanisms can significantly influence bacterial diversity and metabolic capabilities. Phages can protect their bacterial hosts from other predatory phages by integrating prophage elements. A prophage is a latent viral genome that either attaches to or integrates into the bacterial chromosome and replicates alongside the host genome (Canchaya et al., 2003). Prophages are prevalent in bacterial genomes, with estimates suggesting that up to 20% of bacterial genomes harbor detectable lysogenic phages. Consequently, lysogeny may play a substantial role in shaping the adaptation and evolution of microbial communities (Casjens, 2003).

Lysogenic phages can directly influence host evolution by integrating into the bacterial genome. Some integrate at specific loci with minimal disruption and may even confer immunity against superinfection by other phages. The protective mechanisms encoded by such prophages appear to be widespread in nature (Touchon et al., 2016). Previous studies have identified bacterial immune mechanisms, such as the CRISPR-Cas system, which captures exogenous genetic sequences (Seed et al., 2013), and the abortive infection (Abi) system, which induces host cell death to limit phage propagation (Lopatina et al., 2020), as strategies to reduce phage invasion. However, recent evidence suggests that prophage-mediated protection against heterologous phages may be more prevalent than homologous immune mechanisms (Dedrick et al., 2017). Bacteria and their associated phages co-evolve, benefiting from the viral genes embedded within their genomes. This co-evolutionary process enhances bacterial adaptability to changing environments and has significant ecological implications for microbial communities.

Phages drive the evolution of bacterial host populations through horizontal gene transfer. As vectors of horizontal gene transfer, phages mediate gene exchange via generalized and specialized transduction. Upon acquiring horizontally transferred genes, individual bacteria and entire bacterial communities undergo phenotypic changes that influence the evolution of bacterial genomes (Wielgoss et al., 2016). Transduction is increasingly recognized as a key driver of bacterial adaptation to environmental changes. Moreover, the identification of antibiotic resistance genes within isolated phage genomes and virome datasets indicates that phages can act reservoirs for antibiotic resistance genes and facilitate their transfer between bacterial species (Calero-Cáceres et al., 2019). However, this perspective remains controversial, as bacterial contamination of viral samples or the use of low similarity thresholds in predicting antibiotic resistance genes may lead to an overestimation of the abundance of such genes in virome datasets. Nonetheless, several studies have demonstrated that toxin-encoding phages from pathogenic bacteria can convert non-pathogenic bacterial strains into virulent ones through transduction and lysogenization. Phages play a significant role in promoting bacterial evolution by influencing mutation rates. Under selective pressure from lytic phages, bacterial clones with higher mutation rates are more likely to survive. Recent studies have indicated that in more ecologically complex environments, the presence of multiple phages can further accelerate bacterial evolution and enhance the selection of hypermutator strains (Betts et al., 2018).

## 3 The interaction between phages and bacteria

The metaphor “run as hard as you can to stay in the same place” was famously used by the evolutionary biologist Leigh van Valen to propose the “Red Queen Hypothesis,” an influential evolutionary theory suggesting that species must continuously adapt and evolve to survive and pass on their genetic material to future generations (Žliobaitė et al., 2017; Aubier et al., 2020). In natural ecosystems, the interaction between bacteriophages and bacteria exemplifies such a tightly coupled co-evolutionary relationship (Chibani-Chennoufi et al., 2004). Changes in one partner often drive corresponding changes or even extinction in the other. Therefore, mutual adaptation and co-evolution are essential for survival (Barbosa et al., 2013). Bacteriophages have developed multiple survival strategies and transmission mechanisms, exploiting prokaryotic hosts for replication. By infecting bacteria, they influence bacterial competition, maintain microbial diversity, and mediate horizontal gene transfer (Tokuda and Shintani, 2024). Evidence from recent studies indicates that microbial populations are highly dynamic and rapidly evolving. For instance, a longitudinal study of the human gut virome over two and a half years revealed a high turnover rate of bacteriophage lineages, particularly among lytic phages (Shkoporov et al., 2019).

To defend against bacteriophage infection, bacteria have evolved multiple immune systems that resist phage invasion at various stages of the phage life cycle (Hall et al., 2011). Predictably, bacteriophages have also evolved countermeasures to overcome these defenses. This dynamic, combined with the vast diversity of phages, drives the evolution of bacterial immune mechanisms (Gao and Feng, 2023). Consequently, bacteria and bacteriophages have evolved into more resistant (i.e., capable of resisting a broader range of phage genotypes) and more infectious (i.e., capable of infecting a wider array of bacterial genotypes) forms, respectively. This evolutionary pattern is commonly referred to as an “arms race” (Hampton et al., 2020). The ongoing interaction between bacteriophages and bacteria involves continuous development and refinement of defense and counter-defense systems. This long-term co-evolutionary struggle contributes to the complexity of their interactions.

For a bacteriophage to successfully infect a bacterial cell, it must first bind to receptor proteins on the bacterial surface to complete adsorption, followed by disruption of the bacterial membrane to inject its genome. To prevent phage adsorption, bacteria may modify or mask their surface receptors. For example, a mutation in the ompU receptor of *Vibrio cholerae* confers resistance to bacteriophage ICP213 (Seed et al., 2014). Another defense mechanism involves blocking the entry of phage nucleic acids into the bacterial cell. For instance, the Imm and Sp proteins of T4 bacteriophage inhibit translocation of phage nucleic acids across the membrane (Dy et al., 2014). Despite such modifications or mutations in bacterial surface receptors, some bacteriophages can still successfully inject their genetic material. Therefore, bacteria have evolved additional innate immune systems to detect and degrade invading phage nucleic acids. The restriction-modification (RM) system is one of the most well-characterized innate immune mechanisms against phages. It typically consists of methyltransferases and restriction endonucleases. Methyltransferases recognize specific DNA sequences and methylate them, while restriction endonucleases cleave unmethylated DNA at these sites (Dupuis et al., 2013). Bacteria employ RM systems to degrade the nucleic acids of invading bacteriophages, serving as a primary defense mechanism. In countermeasure, bacteriophages have evolved multiple strategies to evade this host-mediated degradation. Some phages minimize the number of restriction sites within their genomes or position these sites too far apart to be effectively recognized by the host’s restriction endonucleases. Others ensure protection of their newly synthesized DNA through methylation, either by hijacking the host’s modification machinery or via self-encoded methyltransferases. Additionally, certain bacteriophages produce hydrolases that specifically target and degrade essential cofactors of restriction enzymes, thereby irreversibly inhibiting their enzymatic activity. These adaptive mechanisms collectively enhance phage survival in RM-protected bacterial hosts (Vasu and Nagaraja, 2013; Labrie et al., 2010).

In addition, bacteria have evolved the adaptive immune system known as the CRISPR-Cas system to resist bacteriophage invasion (Nussenzweig and Marraffini, 2020). CRISPR-Cas immunity is present in approximately 40% of sequenced bacterial genomes and mediates resistance to bacteriophages through three distinct stages: adaptation, expression, and interference. To date, two major classes, six types, and over 30 subtypes of CRISPR-Cas systems have been identified. The first class comprises types I, III, and IV, which are characterized by multi-subunit effector complexes. The second class includes types II, V, and VI, which are defined by single-subunit effector proteins (Koonin and Makarova, 2019). Bacteriophages, in turn, have evolved mechanisms to counteract bacterial adaptive immunity. Anti-CRISPR (Acr) proteins have been identified in some phages, which inhibit the activity of CRISPR–Cas systems—represent one of the most extensive families of natural protein inhibitors characterized to date, with over 90 families employing diverse molecular mechanisms (Wiegand et al., 2020; Davidson et al., 2020; Bondy-Denomy et al., 2018). For example, the anti-CRISPR protein AcrIF25 inhibits the type I-F CRISPR–Cas system by actively disassembling the fully formed effector complex. AcrIF25 specifically targets the core CRISPR RNA-binding components of the complex, which consist of six Cas7 subunits, and sequentially strips them from the RNA scaffold. Structural and biochemical evidence reveals that AcrIF25 removes Cas7 subunits in a stepwise manner, initiating from one end of the complex, without requiring external energy input or enzymatic activity (Trost et al., 2024). If phages successfully bypass the RM and CRISPR-Cas defense systems, bacteria may activate an Abi system as a final defense strategy. This system triggers cell arrest or programmed cell death, thereby halting the phage replication cycle, limiting its spread, and protecting the broader bacterial population (Garb et al., 2022).

**Figure 2:**
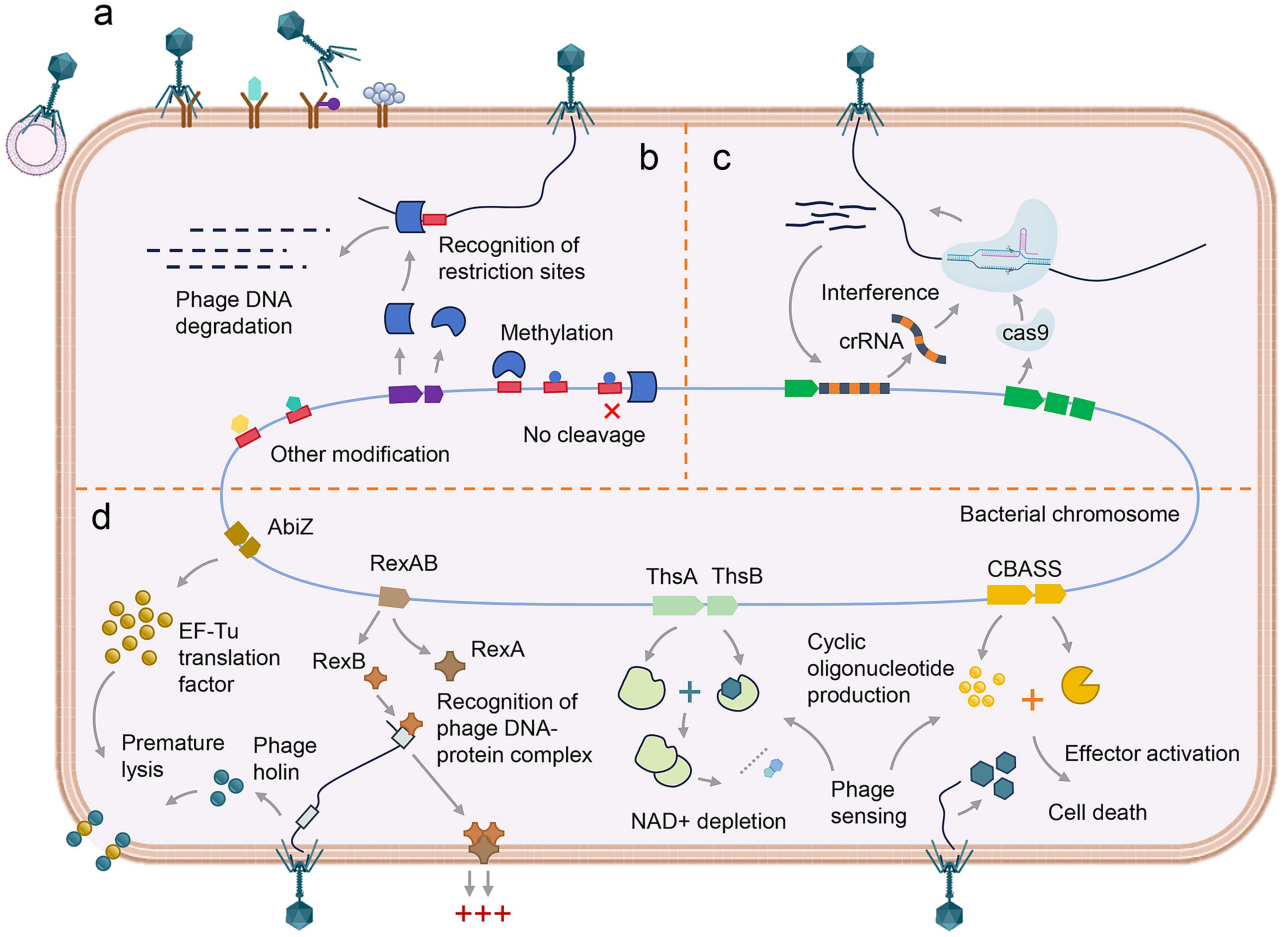
Bacterial defense mechanisms against phage infection. (a) The inhibition of phage adsorption. (b) The degradation of phage DNA mediated by restriction enzymes at specific recognition sites. (c) CRISPR-Cas9-mediated interference, in which crRNA guides the Cas9 nuclease to target and cleave complementary phage DNA. (d) The abortive infection systems, including AbiZ system, RexAB system, ThsAB system and CBASS system, collectively induce cell cycle arrest and programmed cell death.

Overall, the interaction between bacteria and bacteriophages lies at the heart of microbial community ecology and evolution. This interaction is a complex and dynamic process, characterized by a continuous evolutionary arms race. The immune defense of bacteria against bacteriophages involves a coordinated interplay of multiple mechanisms. Bacteria initially employ passive immunity to inhibit phage adsorption and block the entry of bacteriophage DNA. Subsequently, active immune systems—such as the RM and CRISPR-Cas systems—interfere with phage DNA replication and gene expression. In addition, Abi represents a higher-order altruistic defense strategy, in which infected bacterial cells undergo programmed self-destruction prior to the completion of the phage replication cycle, thereby preventing viral spread and safeguarding the survival of neighboring cells within the population (Figure 2). On the other hand, bacteriophages have developed corresponding strategies to overcome bacterial defenses (Yirmiya et al., 2024). Typically, bacteriophages exhibit a higher mutation rate than their bacterial hosts, granting them a significant evolutionary advantage (Pereira-Gómez and Sanjuán, 2015).

## 4 The sequence composition characteristics of phage-bacterial interactions

**Figure 3:**
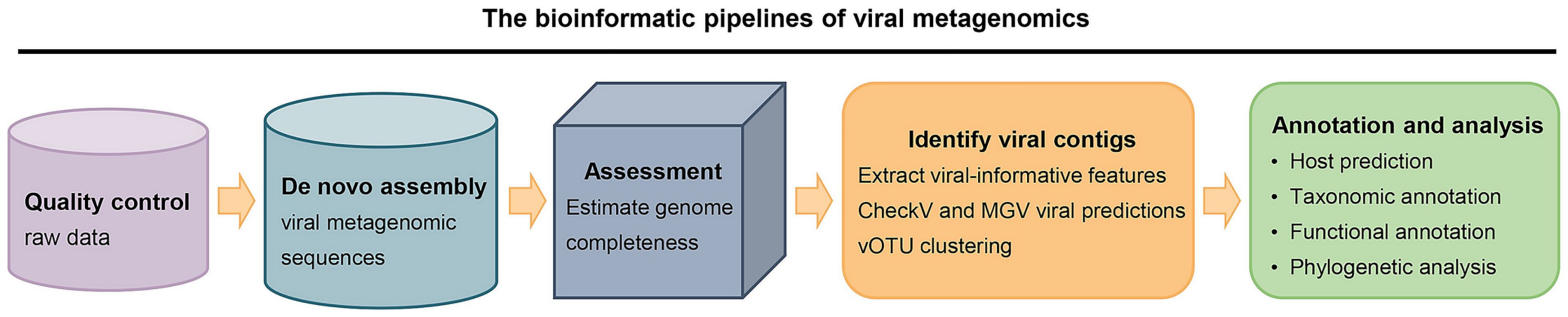
The bioinformatic pipelines of viral metagenomics. (1) Quality control of raw sequencing data; (2) De novo assembly of viral metagenomic sequences; (3) Assessment of genome completeness; (4) Identification of viral contigs through feature extraction, viral gene prediction, and clustering; and (5) Annotation and analysis, encompassing host prediction, taxonomic classification, functional annotation, and phylogenetic analysis. Arrows indicate the directional progression between stages.

The advancement of high-throughput metagenomic sequencing technology has enabled the direct identification of bacteriophages and their hosts from environmental samples without the need for cultivation, offering a powerful tool for comprehensive studies of phage-bacteria interactions (Zhang et al., 2006; Reyes et al., 2010). The analytical workflow of viral metagenomics comprises the following key steps: ① Quality control and preprocessing of raw sequencing data, including the removal of adapter sequences, low-quality reads, and contaminating genomic sequences derived from host organisms such as animals or bacteria; ② *De novo* assembly of viral metagenomic sequences; ③ Assessment of assembly quality using metrics such as contig length, coverage depth, and completeness; ④ Identification of virus-like sequences, followed by taxonomic classification and functional annotation; ⑤ Phylogenetic analysis and host bacterial prediction (Figure 3).

The discovery of anti-CRISPR proteins originated from comparative genomic analysis of bacteriophages exhibiting sensitivity versus resistance to the type I-F CRISPR-Cas system. Genomic similarity facilitates the identification of key genetic differences underlying distinct phenotypes, enabling targeted candidate gene discovery. This strategy was subsequently applied to archaeal systems, leading to the identification of the first archaeal Acr protein (He et al., 2018). In that study, a deletion mutant of the lytic archaeal virus SIRV2 was isolated and its genome sequence was compared with those of wild-type SIRV2 and the closely related, CRISPR-resistant virus SIRV3. Through this comparative approach, the pool of potential Acr candidates was narrowed to three genes. Each gene was individually tested for anti-CRISPR activity, ultimately revealing AcrID1 as a functional inhibitor conferring CRISPR resistance.

This systematic pipeline enables comprehensive characterization of viral communities and their functional potential in complex environments. However, as metagenomic data are culture-independent, they lack the capacity to directly observe phage-host interactions. Fortunately, during the co-evolutionary process between bacteriophages and bacteria, various genomic signals have been left behind that can be used to infer potential host-phage relationships (Versoza and Pfeifer, 2022). Currently, several bioinformatics tools have been developed to predict the likely host range of bacteriophages on a large scale by analyzing shared genomic features resulting from co-evolution (Ahlgren et al., 2017). Although these methods are inherently predictive, they serve as valuable tools for identifying the most promising candidates for experimental validation (Galiez et al., 2017). These candidates can then be used to investigate phage-host recognition, adsorption, infection dynamics, interaction patterns, and lysis efficiency.

Abundance patterns of bacteriophages and bacteria reflect their ecological relationships. The genomes of bacteriophages and their bacterial hosts exhibit temporal and spatial correlations, which apply not only to lysogenic phages integrated into the host genome but also to lytic phages that depend on their hosts for replication (Lu et al., 2021). Bacteriophages can only proliferate in environments where their bacterial hosts are present. Metagenomic data provide snapshots of microbial communities at specific times and locations, enabling the simultaneous identification of co-occurring bacteriophages and their hosts (Mojica et al., 2005). This allows for the establishment of genomic linkages between phages and their hosts. However, the abundance distribution of bacteriophages and their hosts is influenced by multiple factors, including the scale of phage outbreaks, whether the phage is lytic or lysogenic, the presence of host antiviral defense systems, the host range of the phage, and environmental stability (Sun et al., 2024b). Furthermore, bacteriophage and microbial metagenomes are sometimes sequenced separately and subjected to amplification steps to increase yield, which may distort the observed abundance and affect the accuracy of predicted phage-host associations.

Sequence similarity searches are the most direct method for identifying genetic homology and predicting associations between bacteriophages and their bacterial hosts based on genomic sequences (Roslund et al., 2020). These homologous sequences may reflect the acquisition of bacterial DNA by phages during previous infection events. For example, the CRISPR-Cas system can capture exogenous DNA and integrate it into its own spacer arrays (Brouns et al., 2008). Notably, both lytic and lysogenic bacteriophages can acquire and incorporate host genetic material, with selectively advantageous genes being preserved in phage genomes through natural selection (Anantharaman et al., 2016). Gene families that are prone to horizontal gene transfer may be more frequently exchanged between phages and hosts.

In bacteria and archaea, translation selection favors optimal codons for efficient gene expression. Since viral replication relies on the host’s translational machinery, bacteriophages that utilize host-like codons or tRNA isozymes can enhance the efficiency of phage gene translation, which benefits viral replication. Some phages also encode tRNA genes to modify the codon usage bias of their hosts (Robertson et al., 2021; Burman et al., 2024). Additionally, the oligonucleotide frequency patterns used by phages may be shaped by evolutionary pressures to avoid host restriction enzyme recognition sites. Therefore, phages and their bacterial hosts can be linked through similarities in oligonucleotide frequency profiles. The oligonucleotide Hidden Markov Model (HMM) score derived from bacteriophage and bacterial genomes is used to infer potential interactions between bacteriophages and their bacterial hosts. This metric reflects the similarity in oligonucleotide frequency patterns between the host bacterium and the bacteriophage’s nucleic acid sequence. Specifically, this similarity is defined as the maximum likelihood value obtained from HMMs trained on both phage and bacterial genomic sequences. A higher likelihood value indicates greater similarity in oligonucleotide composition, thereby suggesting a higher probability of a biological interaction between the bacteriophage and its bacterial host.

The co-evolutionary process at both molecular and ecological levels has shaped the genomes of bacteriophages and bacteria. The initial interaction between phages and their hosts involves the binding of phage particles to specific receptor molecules on the surface of bacterial cells (Bin Jang et al., 2019). After injecting their genomes into host cells, phages must hijack host metabolism to support efficient phage production. To achieve this, phages have evolved specific proteins that interact with host proteins to inhibit, activate, or redirect their functions, thereby manipulating host cellular machinery to produce new phage progeny (Jin et al., 2025). Using metagenomics, researchers discover that CRISPR systems are widely encoded in diverse bacteriophages, where they function as highly divergent and hypercompact antiviral defense mechanisms. Bacteriophage-encoded CRISPR systems span all six known CRISPR-Cas types, although some lack essential components—indicating potential alternative functional roles or reliance on host-encoded factors for activity. Notably, among the most evolutionarily divergent enzymes identified, Casλ recognizes double-stranded DNA through a uniquely structured CRISPR RNA. Cryo-electron microscopy analysis of the Casλ–RNA–DNA complex reveals a compact bilobed architecture, which demonstrates robust genome-editing activity in both plant and human cells (Al-Shayeb et al., 2022). However, approximately 70% of currently sequenced phage genes encode proteins with unknown functions, and only a limited number of phages have been systematically studied for their molecular interactions with host proteins. Therefore, identifying and characterizing the protein–protein or domain-domain interactions that involved in phage-host interactions remains a significant challenge.

Although numerous computational methods have been developed to predict phage–host interactions, their predictive accuracy remains limited, particularly when relying on a single type of phage-bacteria interaction signal, leading to a significant performance bottleneck. Meanwhile, the exponential growth in virus discovery through viromic studies has created an urgent demand for a comprehensive and user-friendly tool capable of integrating diverse interaction signals for accurate host prediction. However, current tools are constrained by their focus on specific interaction features and dependence on only one type of signal. Moreover, no publicly available web server or standalone software has yet been developed to integrate all known types of phage-bacteria interaction evidence for systematic and holistic prediction of phage–host associations. Therefore, the development of a unified framework that effectively combines multiple biological signals to achieve highly accurate and comprehensive predictions remains an outstanding challenge in the field.

## 5 Conclusions and prospects

Through long-term evolutionary struggle against bacteriophages, bacteria have developed complex immune systems that collectively enhance their survival. The advancement of large-scale genomic and metagenomic sequencing technologies has greatly advanced the understanding of bacteria-phage interaction mechanisms using bioinformatics approaches. By integrating bioinformatics with molecular biology, microbiology, and complementary experimental approaches, researchers have identified a range of novel bacterial defense systems against bacteriophages, significantly advancing our understanding of phage–bacteria interactions. Despite these advances, several challenges remain in the study, particularly regarding the necessity of coupling meta-omics with experimental validation. These challenges represent important directions for future research:

1. Although host prediction is inherently a critical component of any virome analysis pipeline and has been the focus of extensive research and tool development over the past decade, computationally linking uncultured viruses to their bacterial hosts remains a significant challenge.
2. Although significant progress has been made in characterizing individual immune mechanisms, our understanding of how these mechanisms interact and coordinate remains limited. It is still unclear whether a single mechanism acts independently or whether multiple mechanisms operate sequentially or in parallel during phage infection.
3. The high diversity of bacteriophages increases the complexity of studying bacterial immunity. Further research is needed to explore how the same bacterial species selects among different immune mechanisms when facing the same or different phages, as well as the variability in immune response sensitivity.
4. With the increasing availability of large-scale datasets, there is a growing need to apply artificial intelligence (AI) methods to uncover potential associations in bacteria-phage interactions and improve the accuracy of predictive algorithms. The application of AI tools such as AlphaFold to predict novel functions of phage proteins has led to the discovery of a previously unknown mechanism of phage immune evasion.
